# Pak1 overexpression is associated with mild concentric cardiac remodeling and favorable systolic and diastolic functional indices in transgenic mice

**DOI:** 10.3389/fcvm.2026.1858786

**Published:** 2026-09-30

**Authors:** Johnathan Yarbro, Tamayo Kobayashi, Youhua Zhang, Elizabeth Levy, Yuan Huang, Satoru Kobayashi, Qiangrong Liang

**Affiliations:** Department of Biomedical Sciences, New York Institute of Technology, College of Osteopathic Medicine, Old Westbury, NY, United States

**Keywords:** cardiac function, echocardiography, hemodynamics, Pak1, speckle-tracking

## Abstract

**Background:**

p21-activated kinase 1 (Pak1) is a serine/threonine kinase implicated in cardioprotection, calcium handling, and excitation-contraction coupling. However, the effects of Pak1 gain- and loss-of-function on cardiac performance remain incompletely understood. Here, we characterized baseline overall cardiac performance in cardiac-specific Pak1 transgenic (TG) and knockout (KO) mice using advanced echocardiography and invasive hemodynamic assessments.

**Methods:**

Adult wild type (WT), Pak1 TG, and Pak1 KO mice, aged 7–11 months, underwent comprehensive phenotyping, including M-mode echocardiography, pulsed-wave and tissue Doppler imaging, speckle-tracking echocardiography (STE)-based strain analysis, invasive left ventricular pressure measurements, Western blot analysis, and histological evaluation using hematoxylin and eosin and Masson's trichrome staining. In addition, WT and Pak1 TG mice aged 20–22 months were studied to assess persistence of the TG phenotype with aging.

**Results:**

M-mode echocardiography showed that Pak1 TG mice exhibited concentric LV remodeling, characterized by increased wall thickness and reduced chamber dimensions compared with KO and WT controls. Histological analysis revealed greater cardiomyocyte cross-sectional area and width in TG mice than in KO mice, whereas WT values were intermediate and did not differ significantly from TG. Masson's trichrome staining detected no significant difference in myocardial fibrosis between TG and WT hearts under the present sampling conditions. Atrial natriuretic peptide (ANP) and brain natriuretic peptide (BNP) expression was not elevated in TG hearts. TG hearts exhibited higher fractional shortening and increased global longitudinal, radial, and circumferential strain, indicating increased echocardiographic indices of systolic function. Invasive hemodynamics showed higher +*dP*/*dt*_max_ without a significant difference in aortic blood pressure, although these findings do not establish load-independent intrinsic contractility. Diastolic indices also differed, including increased diastolic strain rates, shorter IVRT, lower Tau, and reduced myocardial performance index, whereas *E*/*e*′ was unchanged. Similar remodeling and functional indices were observed in aged TG mice, although the cross-sectional design does not establish preservation with aging. Pak1 KO mice showed modestly reduced systolic indices with preserved diastolic indices.

**Conclusions:**

Pak1 overexpression is associated with concentric LV remodeling and favorable systolic and diastolic functional indices. These findings support further investigation of Pak1-dependent mechanisms and its role in cardiac disease.

## Introduction

Cardiovascular diseases (CVD) are the leading cause of death in the U.S. and globally (1). Coronary heart disease, heart failure (HF), stroke, and hypertension have a pervasive presence, with over half of American adults ≥40 affected by one or more of these conditions (1). The impact of CVD is multifaceted and not only robs individuals of their health but also places a tremendous economic burden on the healthcare system. With 7.7 million American adults currently diagnosed with HF (1), identifying molecular targets that preserve cardiac function under stress is critically needed. Among signaling pathways that regulate cardiac adaptation to stress, Rho-GTPase effectors have emerged as promising therapeutic targets due to their roles in cytoskeletal remodeling and contractile function (2).

p21-activated kinase 1 (Pak1) is a serine/threonine kinase activated by Rho-GTPases (Cdc42/Rac1) that plays an important regulatory role in a variety of cellular processes including cytoskeletal dynamics, cell growth and proliferation, and cardiovascular homeostasis. Pak1 confers robust cardioprotection against mechanical and metabolic stress, including pressure-overload induced hypertrophy (3–6) and ischemia-reperfusion injury (7), while attenuating age-related diastolic dysfunction (8) and oxidative damage (9). Beyond structural protection, Pak1 maintains cardiac electrical stability (10), calcium homeostasis (11), and excitation-contraction coupling through downstream effectors including protein phosphatase 2A (PP2A) and various sarcomeric proteins (9, 12, 13).

Collectively, these findings establish Pak1 as a multifaceted regulator of cardiac physiology and stress adaptation. However, a comprehensive *in vivo* characterization of how Pak1 expression levels influence baseline cardiac structure and function remains lacking. To our knowledge, detailed echocardiographic phenotyping of transgenic Pak1 overexpression (TG) has never been reported, and existing studies of Pak1 knockout (KO) mice are largely limited to basic M-mode and/or Doppler metrics, primarily under stress or aging conditions (3, 4, 8, 14). Consequently, it remains unclear whether Pak1 overexpression drives adaptive or maladaptive remodeling, and whether Pak1 deficiency alone is sufficient to impair cardiac function under basal, non-stressed conditions.

These questions have gained additional translational significance with the recent development of direct allosteric Pak1 activators that demonstrate therapeutic efficacy in models of cardiac hypertrophy, underscoring the importance of defining how altered Pak1 signaling impacts baseline cardiac structure and function (15). Notably, conventional M-mode and Doppler indices have well-recognized limitations in murine studies, including reduced sensitivity for detecting early contractile dysfunction and technical challenges in accurately assessing diastolic function (16–21). In contrast, speckle-tracking echocardiography (STE) offers a more sensitive and less geometry-dependent assessment of myocardial deformation, enabling comprehensive evaluation of both systolic and diastolic mechanics across longitudinal, radial, and circumferential axes.

Accordingly, we performed the first comprehensive echocardiographic and hemodynamic characterization of Pak1 TG and Pak1 KO mice under baseline conditions, integrating STE-derived strain analysis with conventional M-Mode, Doppler imaging, and invasive pressure catheterization. We further examined whether the Pak1 TG phenotype persists into advanced age, an important consideration for understanding the long-term functional relevance of Pak1 signaling.

## Materials and methods

### Animals

All animal procedures were approved by the Institutional Animal Care and Use Committee of New York Institute of Technology. Studies were conducted in male and female adult mice on a C57BL/6 background. Wild-type C57BL/6 mice served as controls. The Pak1 KO line was generated via traditional embryonic stem cell targeted deletion, resulting in a constitutive whole-body KO (22). The cardiac-specific Pak1 TG overexpressing mouse line was generated by pronuclear injection of a construct in which a constitutively active, phosphomimetic human Pak1-T423E cDNA was placed under control of the α-myosin heavy chain (α-MHC) promoter to restrict transgene expression to cardiomyocytes. Genotypes were determined by polymerase chain reaction analysis of tail biopsies and confirmed post-mortem via western blot using a Pak1 Antibody (Cell Signaling #2602). Mice were housed in the same animal room in a temperature-controlled environment on a 12-h light/dark cycle with *ad libitum* access to food and water. Most studies were performed in adult mice aged 7–11 months. In addition, WT and Pak1 TG mice aged 20–22 months were studied to determine whether the TG phenotype is maintained with aging. The specific ages used in each experiment are indicated in the corresponding tables and figure legends. All procedures were performed by an investigator blinded to genotype.

### Echocardiography

To assess cardiac geometry and function, transthoracic echocardiograms were performed using a VisualSonics Vevo 3100 imaging system (FUJIFILM VisualSonics Corporation, WA, USA), with an MX550 transducer (32–40 MHz frequency), and analyzed with Vevo LAB software, version 5.11.1. Imaging acquisition and analysis were performed broadly in accordance with the guidelines of the European Society of Cardiology (ESC) (23). Mice were first placed into an induction chamber for anesthesia with 3% isoflurane and 1 L/min O_2_. They were then placed on the Vevo 3100 imaging station which has electrode pads for ECG readings and is heated to maintain the body temperature of the mouse at 37 ℃. Anesthesia was maintained through a nose-cone at 1%–2% isoflurane (and 1 L/min O_2_) throughout the procedure, with minor adjustments to isoflurane concentration to maintain heart rate between 300 and 500 bpm. Immediately following hair removal using Nair, images for mice were obtained in the following order: parasternal long-axis (LAX) B-mode, parasternal short-axis (SAX) B-mode, SAX M-Mode, apical four-chamber (AP4C) pulsed-wave Doppler, AP4C tissue Doppler.

M-mode images were acquired from a SAX view at the mid papillary level to measure LV wall and chamber dimensions. Values were averaged over 3–5 consecutive cycles. Parameters reported from M-mode measurements include in systole (s) and diastole (d): LV internal diameter (LVID), LV anterior wall thickness (LVAW), LV posterior wall thickness (LVPW), relative wall thickness (LVAWd + LVPWd)/(LVIDd) and fractional shortening ((LVIDd-LVIDs)/(LVIDd) × 100). Volumetric measurements obtained via M-mode were not reported, as recommended in the ESC guidelines (23), as volume extrapolation is not accurate.

Pulsed wave Doppler (PW) and tissue Doppler (TD) imaging were acquired from the AP4C view to assess diastolic parameters. For PW, transmitral inflow velocities were recorded by setting the sample gate in the mitral orifice close to the mitral leaflets, with the doppler beam parallel to the inflow direction. For TD, the sample gate was placed at the septal mitral annulus. Myocardial performance index (MPI) (25), a Doppler derived measure of global cardiac function, was calculated from PW metrics (IVRT + IVCT)/(AET). All measurements were performed with the operator blinded to genotype.

### Speckle tracking echocardiography (STE)

B-mode images were acquired from LAX (longitudinal strain) and SAX (circumferential and radial strain) views. All images were stored in cine loops consisting of 300 frames with a frame rate of >230 fps for LAX images and >300 fps for SAX images. Images were analyzed using Vevo Strain 2.0 Software. Endocardial and epicardial borders were traced in end-systole and end-diastole, then checked across all cycles (at least 3 for each mouse) to ensure optimal tracking. Endocardial values were used for all strain and strain rate analyses. Global peak systolic strain (%) represents Lagrangian contour-based deformation across the entire endocardial border: Strain = [(endocardial length at end systole − endocardial length at end diastole)/(endocardial length at end diastole)]. Global strain rate (s^−1^) represents the temporal derivative of strain (Δ strain/Δ time), computed by Vevo software across the full contour. Peak systolic and early diastolic strain rates were extracted from the software-reported strain-time curves. All measurements were performed with the operator blinded to genotype.

### Left ventricular hemodynamic and blood pressure measurements

Left ventricular (LV) hemodynamic parameters were obtained via catheterization using a 1.2-F Transonic SciSense pressure catheter (Transonic SciSense Inc, London, Ontario, Canada). Under isoflurane (3%) anesthesia, the right carotid artery was isolated, cannulated, and the catheter's tip was advanced retrograde through the aorta into the LV. The isoflurane was then reduced and maintained at 1.5%. After 15 min of stabilization, the following parameters were measured: heart rate, LV peak systolic pressure, LV end-systolic and end-diastolic pressure, Tau, and positive/negative change in pressure over time (*dP*/*dt*). The catheter was then slightly withdrawn, so the tip was in the aorta to record arterial blood pressure. The LV hemodynamic and blood pressure data were acquired and analyzed via the iWorx LabScribe software, version 26.0101. All measurements were performed with the operator blinded to genotype.

### Tissue harvest, microscopy, histology, and trichrome staining

Mice were euthanized by anesthetic overdose with 4% isoflurane delivered in 1 L/min O₂ and maintained until respiration ceased, followed by thoracotomy and rapid heart excision. Hearts were rinsed briefly in PBS, blotted dry on tissue paper, the atria and great vessels were removed, and ventricles were weighed. A transverse tissue block near the mid-ventricle was collected and fixed in 4% paraformaldehyde in phosphate-buffered saline for 72 h at 4 °C. Fixed tissue was then dehydrated, embedded in paraffin, and sectioned along the ventricular short axis at a thickness of 5 μm. Sections were stained with hematoxylin and eosin (H&E) for morphometric analysis and Masson's trichrome to evaluate myocardial fibrosis. Whole-slide brightfield images were acquired using a ZEISS Axioscan 7 slide scanner at 40× magnification. Morphometric analysis was performed using Fiji (v1.54p) by an operator blinded to genotype.

For histological analysis, eight animals were used per group. The whole cardiac cross sections were stained. Due to technical difficulties and time constraints, tissue sections could not be collected at exactly identical anatomical planes. In each whole section, cardiomyocytes were selected based on prespecified criteria: a nearly round cellular profile, clearly defined borders, and a visible centrally located nucleus. Fifty cardiomyocytes were measured per animal across multiple fields on each section, with each animal not individual cells treated as the experimental unit for statistical analysis. Cardiomyocyte cross-sectional area (CSA) and width were determined by manual tracing. Screenshots of selected cells were collected and reviewed throughout the selection process to ensure that all analyzed cells met the predefined inclusion criteria. All measurements were performed with the operator blinded to genotype.

Myocardial fibrosis was quantified from Masson's trichrome-stained sections by measuring the collagen-positive area using Fiji (v1.54p). After image acquisition and background correction, a color threshold was set to highlight collagen fibers, and the Measure tool was used to calculate the percentage of positive area. Collagen-rich regions were segmented by thresholding blue-stained areas. The collagen-positive area was normalized to the total myocardial area within each section, excluding any non-myocardial spaces, with fibrosis expressed as the percentage of collagen-positive area relative to total tissue area using the following equation:

$$\mathrm{Fibrosis}\;(\text{\%})=\frac{\mathrm{Collagen}-\mathrm{positive}\;\mathrm{area}}{\mathrm{Total}\;\mathrm{tissue}\;\mathrm{area}}\times 100$$

### Western blot analysis

Protein expression was assessed by immunoblotting using standard procedures. Heart tissues were homogenized in ice-cold Cell Lysis buffer (# 9803S, Cell Signaling Technology) supplemented with protease (# 1861279, Thermo Fisher Scientific) and phosphatase inhibitors (# P0044, Millipore Sigma). Lysates were clarified by centrifugation at 12,000×*g* for 15 min at 4 °C, and protein concentrations were determined using a BCA assay (# 23227, Thermo Fisher Scientific).

For collagen I and III detection, 20 µg of protein per lane was resolved on 7% SDS-PAGE gels at 75 V for 15 min through the stacking gel, followed by 100 V for 1.5 h for separation. Proteins were transferred onto PVDF membranes (# 1620177, Bio-Rad) using the wet transfer method at 30 V for 16 h at 4 °C. For ANP and BNP detection, 15 µg of protein per lane was resolved on 10% SDS-PAGE gels at 80 V for 20 min through the stacking gel, followed by 120 V for 1 h for separation, and transferred using the Turbo transfer method (7 min).

Membranes were blocked for 1 h at room temperature in TBST (Tris -buffered saline with 0.1% Tween-20) containing 5% BSA (# A9647, Sigma-Aldrich), then incubated overnight at 4 °C with primary antibodies diluted in blocking buffer. The following primary antibodies were used: Pak1 (1:1,000, #2602, Cell Signaling Technology), α-Actinin (1:5,000, #3134, Cell Signaling Technology), GAPDH (1:50,000, #2118, Cell Signaling Technology), COL3A1 (1:5,000, sc-271249, Santa Cruz Biotechnology), ANP (1:5,000, sc-515701, Santa Cruz Biotechnology), BNP (1:5,000, PA5-96084, Thermo Fisher Scientific), and COL1A1 (1:5,000, MA1-26771, Thermo Fisher Scientific). Additional details regarding reagents and antibodies are provided in the supplemental materials.

After washing three times with TBST, membranes were incubated for 1 h at room temperature with HRP-conjugated secondary anti-rabbit antibodies (1:10,000 in blocking buffer). Immunoreactive bands were visualized using enhanced chemiluminescence (ECL; #TMA-6, Lumigen) and imaged with an Amersham Imager 680. GAPDH and α-Actinin served as loading controls, both of which showed stable expression across WT, TG, and KO hearts. Densitometric quantification of target and loading-control bands was performed using Fiji (v1.54p), and the relative abundance of each target protein was expressed as the ratio to the corresponding loading control.

### Statistics

Statistical analyses were performed using GraphPad Prism (10.6.1). All datasets were tested for normal distribution using the Shapiro–Wilk test. For comparisons among three groups, homogeneity of variance was evaluated with Bartlett's test. When assumptions of normality and equal variance were met, one-way ANOVA was used followed by Tukey's *post hoc* test for pairwise comparisons. When either the Shapiro–Wilk or Bartlett's test indicated a significant departure from assumptions, the Kruskal–Wallis test was used with Dunn's test for pairwise comparisons. Of all our data, only 2 parameters within the invasive hemodynamic data required Kruskal–Wallis test and are indicated in Table 1. For two-group comparisons between aged WT and TG mice, Welch's *t*-test was used in each case to test differences. A *p*-value <0.05 was used to determine significance for all statistical tests.

**Table 1:** Hemodynamic parameters in WT, Pak1 KO, and Pak1 TG mice (mean ± SD). *n* = 7 per group. Overall *P* values from one-way ANOVA unless other-wise noted.

Parameter	WT	KO	TG	*P* value
Baseline characteristics				
Age (days)	314*.*1 ± 35*.*71	238*.*6 ± 6*.*705*	333*.*4 ± 70*.*88^†^	**0**.**0008**^‡^
BW (g)	24*.*23 ± 1*.*276	21*.*86 ± 1*.*170*	24*.*33 ± 2*.*019^†^	**0**.**0113**
VW (mg)	95*.*66 ± 8*.*598	95*.*99 ± 8*.*211	96*.*16 ± 5*.*771	0.9923
VW/BW (mg/g)	3*.*947 ± 0*.*2696	4*.*390 ± 0*.*2647*	3*.*968 ± 0*.*2827^†^	**0**.**0107**
LV hemodynamics				
Heart rate (bpm)	390*.*0 ± 37*.*05	373*.*9 ± 27*.*79	443*.*1 ± 32*.*08^*^^,^^†^	**0**.**0023**
LVSP (mmHg)	104*.*9 ± 8*.*795	105*.*4 ± 4*.*569	101*.*6 ± 6*.*772	0.5433
LVESP (mmHg)	100*.*9 ± 7*.*811	100*.*9 ± 4*.*863	98*.*09 ± 6*.*851	0.6618
LVEDP (mmHg)	9*.*836 ± 3*.*024	10*.*53 ± 1*.*483	7*.*500 ± 1*.*498^†^	**0**.**0395**
+*dP*/*dt*max (mmHg/s)	6,942 ± 942*.*7	6,680 ± 718*.*0	7,878 ± 576*.*5^†^	**0**.**0214**
−*dP*/*dt*min (mmHg/s)	−5,478 ± 1,117	−5,341 ± 682*.*9	−6,134 ± 834*.*4	0.2362
*τ* (ms)	10*.*27 ± 2*.*254	10*.*59 ± 1*.*711	8*.*940 ± 0*.*5226^†^	**0**.**0167**^‡^
Aortic blood pressure				
Systolic BP (mmHg)	104*.*8 ± 9*.*609	104*.*2 ± 7*.*283	98*.*85 ± 7*.*768	0.3576
Diastolic BP (mmHg)	73*.*90 ± 7*.*467	73*.*49 ± 5*.*299	72*.*16 ± 5*.*042	0.8545
Mean BP (mmHg)	87*.*96 ± 8*.*223	87*.*66 ± 5*.*361	84*.*52 ± 6*.*360	0.6247

BW, body weight; VW, ventricular weight; LV, left ventricle; LVSP, LV peak systolic pressure; LVESP, LV end-systolic pressure; LVEDP, LV end-diastolic pressure; *dP*/*dt*max and *dP*/*dt*_min_, maximum and minimum rate of LV pressure change; *τ* (tau), isovolumic relax-ation time constant (Weiss method); BP, blood pressure.

Bold values indicate significant *P*-values from one-way ANOVA.

* *P* *<* 0*.*05 vs. WT (*post-hoc* multiple comparisons).

† *P* *<* 0*.*05 vs. KO (*post-hoc* multiple comparisons).

‡ Kruskal–Wallis test; all other rows by one-way ANOVA.

## Results

### Baseline characteristics and cardiac morphometry of WT, Pak1 TG, and Pak1 KO mice

Pak1 protein levels were elevated by about 7-fold in Pak1 TG mice relative to WT (Figure 1A) and undetectable in KO mice (Figure 1B). Age (∼7 months), bodyweight (BW) (∼25 grams), and sex (6 males, 2 females per group) were comparable among groups (Table 2). While absolute ventricular weight (VW) trended higher in TG mice relative to WT (*p* = 0.16) and KO (*p* = 0.10), these differences did not reach statistical significance (*p* = 0.08). However, VW normalized to BW differed significantly across groups by one-way ANOVA (*p* = 0.04) (Table 2, Figure 2A). *Post-hoc* pairwise comparison showed modest elevations in KO (*p* = 0.08) and TG (*p* = 0.053) relative to WT, though they remained just above the threshold for significance after correction.

**Figure 1:**
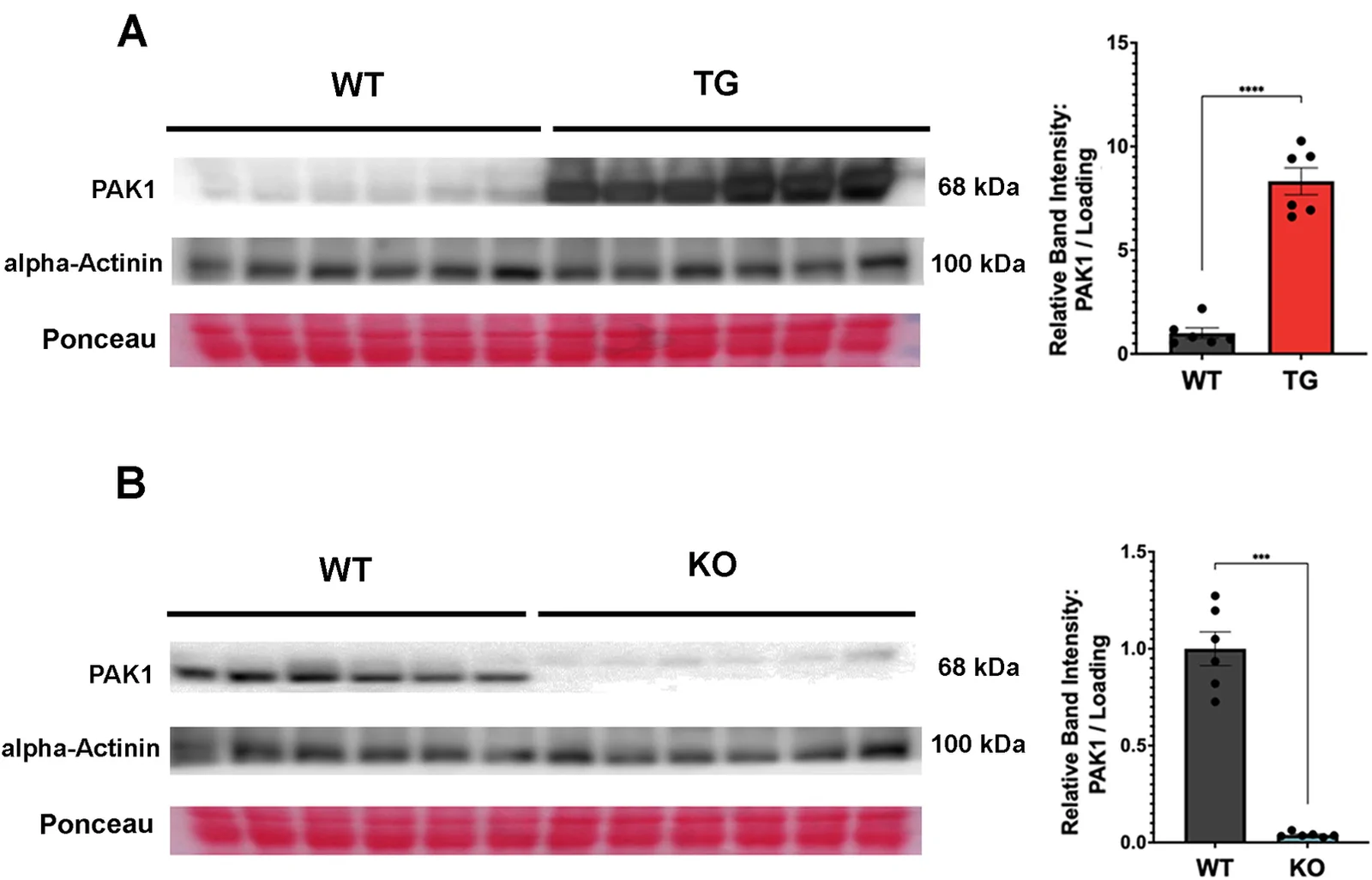
Immunoblot confirmation of cardiac-specific Pak1 transgenic (TG) overexpression and knockout (KO) in mouse hearts. Cardiac tissues from Pak1-TG and KO mice were analyzed by Western blotting. **(A)** Comparison of Pak1-TG vs. wild-type (WT) hearts. **(B)** Comparison of Pak1-KO vs. WT hearts. Representative immunoblots are shown on the left, with corresponding densitometric quantifications on the right. α-Actinin and Ponceau S staining served as loading controls. *n* = 6 per group. Data are presented as mean ± SD. Statistical comparisons were performed using one-way ANOVA followed by Tukey's *post hoc* test. *p < 0.05; ****p < 0.0001.

**Table 2:** Echocardiographic M-mode, Doppler, and speckle-tracking strain pa-rameters in WT, Pak1 KO, and Pak1 TG mice (mean ± SD). *n* = 8 per group. Overall *P* values from one-way ANOVA.

Parameter	WT	KO	TG	*P* value
Baseline characteristics and morphological parameters				
Age (days)	206*.*0 ± 61*.*99	176*.*8 ± 21*.*93	229*.*4 ± 62*.*57	0.1569
BW (g)	26*.*06 ± 3*.*486	23*.*48 ± 1*.*978	26*.*70 ± 3*.*794	0.1251
VW (mg)	104*.*90 ± 14*.*71	103*.*3 ± 9*.*192	118*.*10 ± 16*.*38	0.0829
VW/BW	4*.*040 ± 0*.*4201	4*.*399 ± 0*.*1583	4*.*433 ± 0*.*3126	**0**.**0387**
CM CSA (µm^2^)	250*.*8 ± 39*.*28	213*.*8 ± 22*.*49	270*.*0 ± 44*.*16^†^	**0**.**0151**
CM Width (µm)	18*.*67 ± 1*.*347	17*.*44 ± 0*.*9365	19*.*93 ± 1*.*760^†^	**0**.**0068**
M-mode parameters				
Heart rate (bpm)	352*.*9 ± 31*.*69	396*.*6 ± 35*.*05	381*.*0 ± 54*.*17	0.1272
FS (%)	25*.*75 ± 1*.*849	20*.*84 ± 2*.*561*	41*.*45 ± 4*.*464^*^^,^^†^	<**0***.***0001**
LVID;s (mm)	2*.*936 ± 0*.*1412	3*.*212 ± 0*.*2059*	2*.*103 ± 0*.*2576^*^^,^^†^	<**0***.***0001**
LVID;d (mm)	3*.*957 ± 0*.*2414	4*.*055 ± 0*.*1647	3*.*584 ± 0*.*2436^*^^,^^†^	**0**.**0008**
LVAW;s (mm)	1*.*122 ± 0*.*1958	1*.*015 ± 0*.*07058	1*.*479 ± 0*.*1161^*^^,^^†^	<**0***.***0001**
LVAW;d (mm)	0*.*8291 ± 0*.*1595	0*.*7086 ± 0*.*05278	0*.*8957 ± 0*.*0951^†^	**0**.**0099**
LVPW;s (mm)	1*.*039 ± 0*.*08439	0*.*8772 ± 0*.*1524	1*.*348 ± 0*.*1491^*^^,^^†^	<**0***.***0001**
LVPW;d (mm)	0*.*6969 ± 0*.*05474	0*.*656 ± 0*.*1265	0*.*8408 ± 0*.*1379^*^^,^^†^	**0**.**0090**
RWT	0*.*3875 ± 0*.*06097	0*.*3376 ± 0*.*04248	0*.*4868 ± 0*.*06453^*^^,^^†^	<**0***.***0001**
Doppler parameters				
*E* wave (mm/s)	521*.*7 ± 78*.*27	506*.*1 ± 72*.*58	619*.*9 ± 49*.*29^*^^,^^†^	**0**.**0059**
*A* wave (mm/s)	388*.*1 ± 64*.*12	331*.*5 ± 61*.*70	401*.*8 ± 69*.*86	0.0973
*E*/*A*	1*.*361 ± 0*.*2006	1*.*557 ± 0*.*2423	1*.*586 ± 0*.*3179	0.1897
*e*′ (mm/s)	−16*.*34 ± 1*.*912	−16*.*29 ± 2*.*747	−20*.*42 ± 3*.*448^*^^,^^†^	**0**.**0096**
*E*/*e*′	31*.*84 ± 1*.*964	31*.*49 ± 4*.*603	31*.*22 ± 6*.*395	0.9655
*s*′ (mm/s)	13*.*94 ± 1*.*809	14*.*58 ± 2*.*886	18*.*26 ± 2*.*604^*^^,^^†^	**0**.**0044**
IVCT (ms)	16*.*84 ± 3*.*737	16*.*63 ± 3*.*667	14*.*06 ± 1*.*343	0.1649
AET (ms)	59*.*90 ± 9*.*254	58*.*56 ± 5*.*869	57*.*51 ± 8*.*458	0.8372
IVRT (ms)	27*.*38 ± 3*.*418	27*.*20 ± 5*.*567	20*.*13 ± 2*.*265^*^^,^^†^	**0**.**0019**
MPI	0*.*7471 ± 0*.*1049	0*.*7505 ± 0*.*1080	0*.*6018 ± 0*.*06404^*^^,^^†^	**0**.**0065**
Speckle-tracking echocardiography				
Global strain				
GLS (%)	−15*.*45 ± 1*.*296	−11*.*83 ± 1*.*022*	−22*.*87 ± 1*.*951^*^^,^^†^	<**0***.***0001**
GRS (%)	29*.*39 ± 2*.*562	25*.*57 ± 2*.*916*	42*.*61 ± 1*.*328^*^^,^^†^	<**0***.***0001**
GCS (%)	−21*.*49 ± 0*.*8938	−18*.*82 ± 2*.*184*	−35*.*98 ± 2*.*121^*^^,^^†^	<**0***.***0001**
Systolic peak strain rate				
Longitudinal (s^−1^)	−4*.*025 ± 0*.*3576	−3*.*363 ± 0*.*1408	−6*.*275 ± 0*.*8396^*^^,^^†^	<**0***.***0001**
Radial (s^−1^)	5*.*725 ± 0*.*7206	5*.*325 ± 0*.*6453	7*.*763 ± 1*.*184^*^^,^^†^	<**0***.***0001**
Circumferential (s^−1^)	−5*.*575 ± 0*.*5574	−5*.*238 ± 0*.*5263	−10*.*54 ± 0*.*9591^*^^,^^†^	<**0***.***0001**
Diastolic peak strain rate				
Longitudinal (s^−1^)	4*.*663 ± 0*.*6046	4*.*013 ± 0*.*9125	6*.*050 ± 0*.*8367^*^^,^^†^	**0**.**0002**
Radial (s^−1^)	−6*.*150 ± 1*.*372	−5*.*513 ± 0*.*9311	−8*.*838 ± 1*.*996^*^^,^^†^	**0**.**0005**
Circumferential (s^−1^)	6*.*213 ± 0*.*8543	5*.*663 ± 1*.*068	12*.*04 ± 1*.*727^*^^,^^†^	<**0***.***0001**

BW, bodyweight; VW, ventricular weight; CM, cardiomyocyte; CSA, mean cross-sectional area; FS, fractional shortening; LVID;s, LV internal diameter in systole; LVID;d, LV internal diameter in diastole; LVAW;s, LV anterior wall thickness in systole; LVAW;d, LV anterior wall thickness in diastole; LVPW;s, LV posterior wall thickness in systole; LVPW;d, LV posterior wall thickness in diastole; RWT, relative wall thickness; IVCT, isovolumetric contraction time; AET, aortic ejection time; IVRT, isovolumetric relaxation time; MPI, myocardial performance index; GLS, global longitudinal strain; GRS, global radial strain; GCS, global circumferential strain.

Bold values indicate significant *P*-values from one-way ANOVA.

* *P* *<* 0*.*05 Column vs. WT (*post-hoc* multiple comparisons).

† *P* *<* 0*.*05 TG vs. KO (*post-hoc* multiple comparisons).

**Figure 2:**
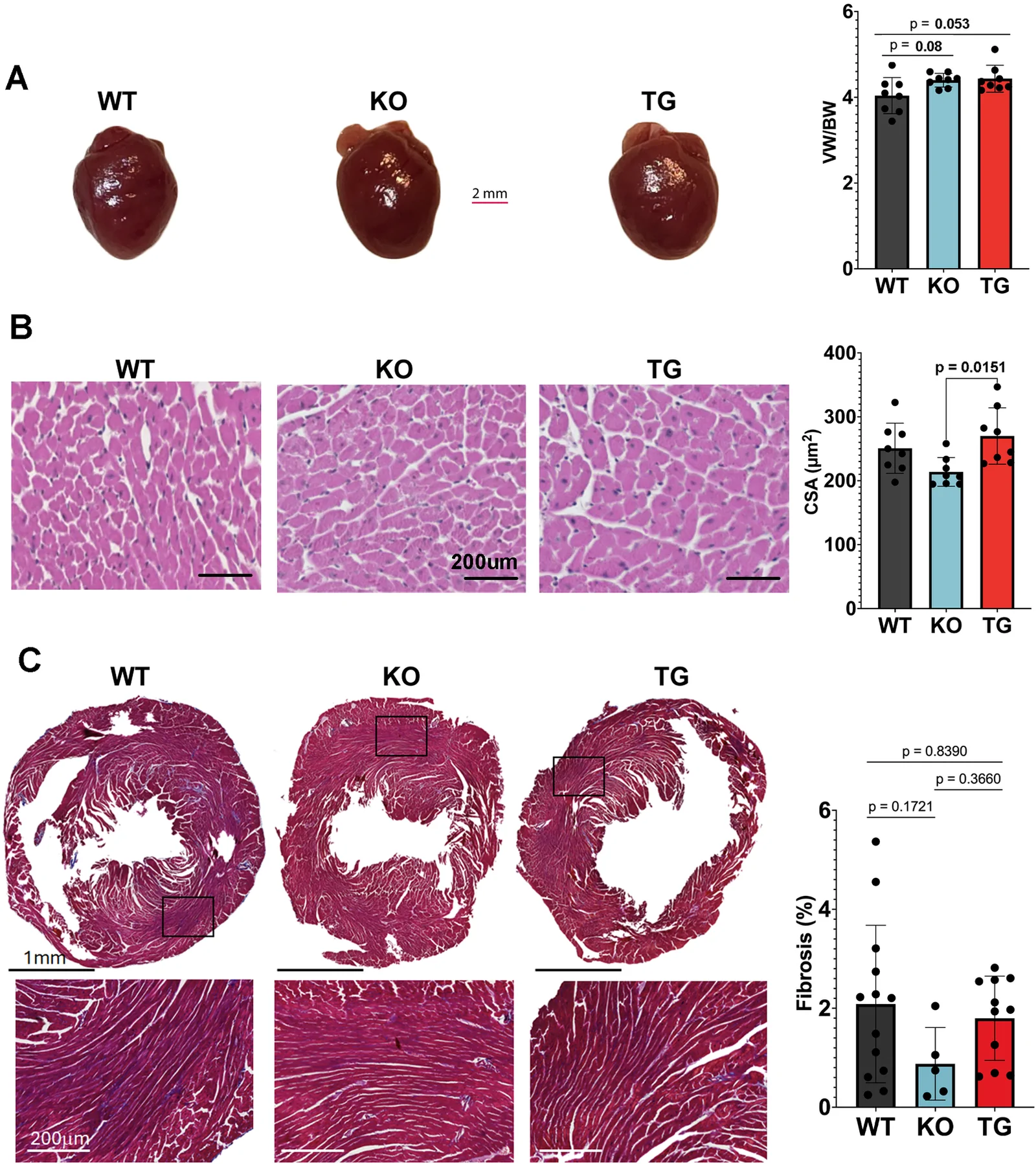
Baseline cardiac morphology and histological characterization of 7-month old WT, PAK1-TG, and Pak1-KO mice. **(A)** Representative gross heart images (left) and ventricular weight normalized to body weight (VW/BW; right). **(B)** Representative hematoxylin and eosin (H&E)-stained left ventricular sections (left) and quantification of cardiomyocyte cross-sectional area (CSA; right). Eight animals were used per group. 50 cardiomyocytes were measured per animal across multiple fields per section, with each animal not individual cells treated as the experimental unit for statistical analysis. **(C)** Representative Masson's trichrome-stained sections from 11-month-old mice (left) and quantification of myocardial fibrosis (right) (WT, *n* = 13; TG, *n* = 11; KO, *n* = 5). Sections were not necessarily collected at identical planes. Scale bars: 2 mm for gross heart images and 200 μm for H&E and Masson's trichrome images. Data are presented as mean ± SD. Statistical comparisons were performed using one-way ANOVA followed by Tukey's *post hoc* test. *p < 0.05.

Consistent with these gross findings, histological analysis showed a concordant trend at the cellular level (representative images shown in Figure 2B). Cardiomyocyte cross-sectional area (CSA) and width differed significantly among groups (*p* = 0.015 and *p* = 0.007, respectively) (Figure 2B, Table 2), with both metrics greater in TG than in KO mice, while WT values were intermediate and not significantly different from either group.

Masson's trichrome staining of tissue sections from 11-month-old mice revealed no significant differences detected under the present sampling and analytical conditions in myocardial fibrosis among Pak1-TG, Pak1-KO, and WT hearts (both *p* > 0.05, Figure 2C), which was associated with the unchanged protein expression of collagen I and collagen IIIA1 (*p* > 0.05, Supplementary Figures S1A,B). Likewise, expression of the pathological hypertrophic marker atrial natriuretic peptide (ANP) was unchanged, whereas brain natriuretic peptide (BNP) expression was unexpectedly reduced in Pak1-TG hearts (*p* < 0.01, Supplementary Figure S1A). Taken together, these results indicate that Pak1 overexpression is well tolerated and does not elicit cardiac pathology.

### Baseline echocardiographic and geometric remodeling

M-mode echocardiography in 7-month-old mice revealed significant differences in left ventricle (LV) geometry among groups (representative images shown in Supplementary Figure S2). Pak1 TG mice demonstrated increased LV anterior wall (LVAW) and posterior wall (LVPW) thickness, accompanied by reduced LV internal diameter (LVID) in both systole and diastole, relative to KO mice (all *p* < 0.01; Figure 3A, Table 2). These structural shifts were also significant relative to WT, with the exception of diastolic LVAW, which was higher in TG but not significant. Relative wall thickness (RWT) was likewise significantly increased in TG relative to WT and KO mice (*p* < 0.01; Figure 3B, Table 2), consistent with a concentric LV geometric phenotype. WT values were intermediate between KO and TG for all dimensional parameters, although differences between KO and WT were modest and reached significance only for systolic LVID. These findings indicate that Pak1 overexpression is associated with a chamber-level remodeling phenotype characterized by increased wall thickness and a reduced LV cavity size under baseline conditions.

**Figure 3:**
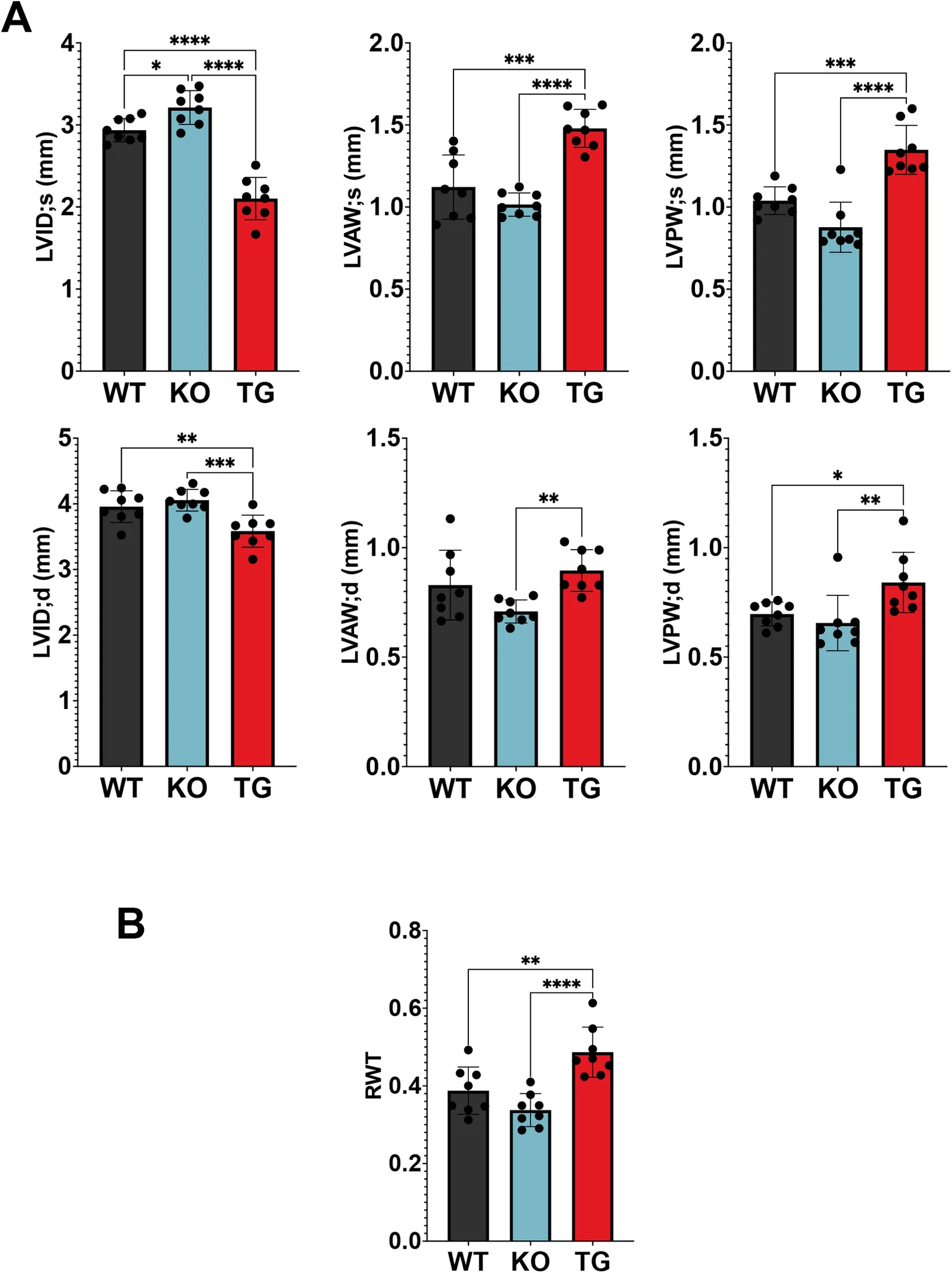
M-mode echocardiography in 7-month-old mice demonstrates a concentric LV geometric phenotype in Pak1 TG mice. **(A)** Relative wall thickness (RWT). **(B)** Quantification of LV anterior wall thickness in systole and diastole (LVAW;s and LVAW;d), LV posterior wall thickness in systole and diastole (LVPW;s and LVPW;d), and LV internal diameter in systole and diastole (LVID;s and LVID;d). *n* = 8 per group. Data are presented as mean ± SD. Statistical comparisons were performed by one-way ANOVA with Tukey's *post hoc* test. *p < 0.05, **p < 0.01, ***p < 0.001, ****p < 0.0001.

### Systolic functional indices

Pak1 TG mice exhibited higher systolic functional indices across multiple echocardiographic measures, whereas KO mice generally showed lower values than WT mice (Figure 4, Table 2). Fractional shortening (FS) was higher in TG mice (41.45 ± 4.46%) than in WT (25.75 ± 1.85%) and KO mice (20.84 ± 2.56%) (Figure 4A). Similarly, global longitudinal strain (GLS) was greater in TG mice (−22.87 ± 1.95%) and lower in KO mice (−11.83 ± 1.02%) compared with WT (−15.45 ± 1.30%) (Figure 4B, Table 2; representative GLS images in Supplementary Figure S3). Global radial and circumferential strain showed similar patterns (Figures 4C,D; Table 2), with representative STE videos provided in Supplementary Video S1. Systolic peak strain rates across all three axes were higher in TG mice than in WT and KO mice (all *p* < 0.0001), whereas KO values did not differ significantly from WT. Tissue Doppler systolic velocity (*s*′) was also higher in TG mice than in WT and KO mice (*p* = 0.004; Table 2). Overall, Pak1 overexpression was associated with higher systolic functional indices, whereas Pak1 deletion was associated with modestly lower systolic indices under baseline conditions.

**Figure 4:**
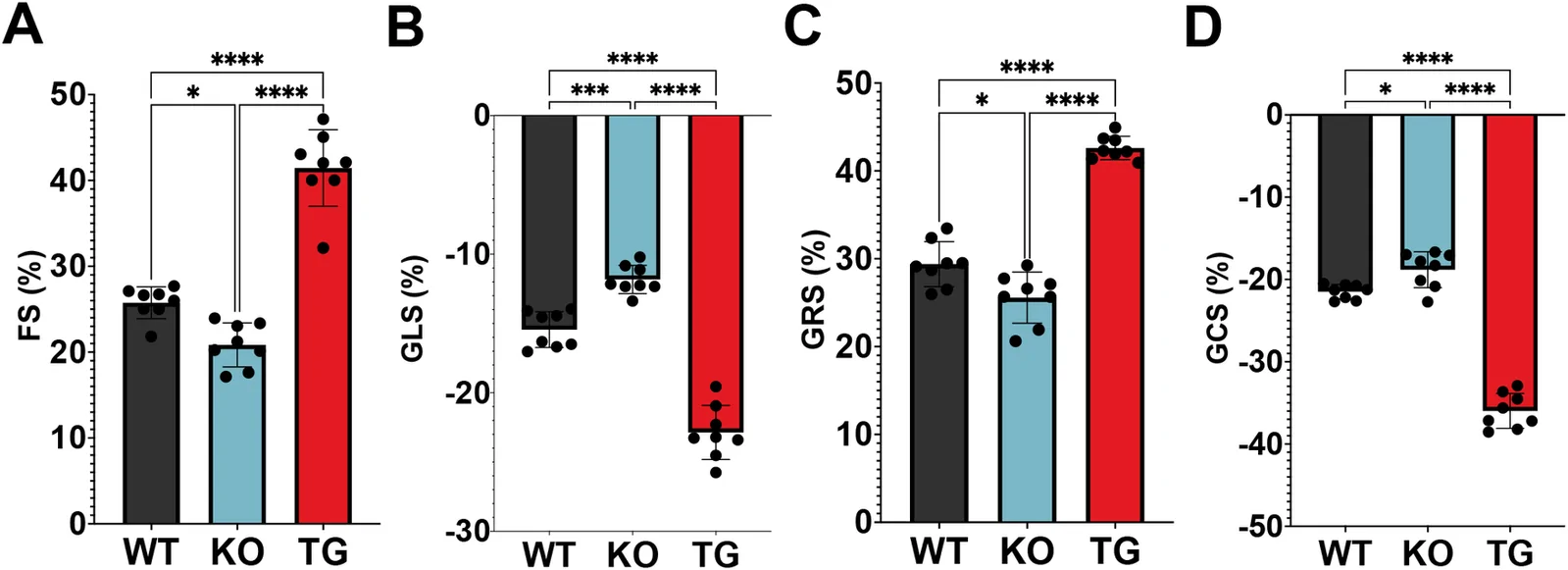
Pak1 overexpression enhances systolic function in 7-month-old mice. Speckle-tracking echocardiographic images for longitudinal strain analysis were performed in WT, Pak1 KO, and Pak1 TG mice. **(A)** Fractional shortening (FS). **(B)** Global longitudinal strain (GLS). **(C)** Global radial strain (GRS). **(D)** Global circumferential strain (GCS). *n* = 8 per group. Data are presented as mean ± SD. Statistical comparisons were performed by one-way ANOVA with Tukey's *post hoc* test. *p < 0.05, **p < 0.01, ***p < 0.001, ****p < 0.0001.

### Diastolic function and global myocardial performance

Pak1 TG mice exhibited differences in multiple diastolic functional indices, whereas WT and KO mice were largely similar. Mitral E-wave velocity and early diastolic tissue velocity (*e*′) were higher in TG mice than in WT and KO mice (both *p* < 0.01), while *A*-wave velocity and *E*/*A* ratio did not differ among groups (Table 2). The *E*/*e*′ ratio was unchanged across genotypes (*p* = 0.97) (Figure 5D, Table 2). Diastolic peak strain rates were higher in TG mice across longitudinal, radial, and circumferential axes (all *p* < 0.001; Figures 5A–C, Table 2).

**Figure 5:**
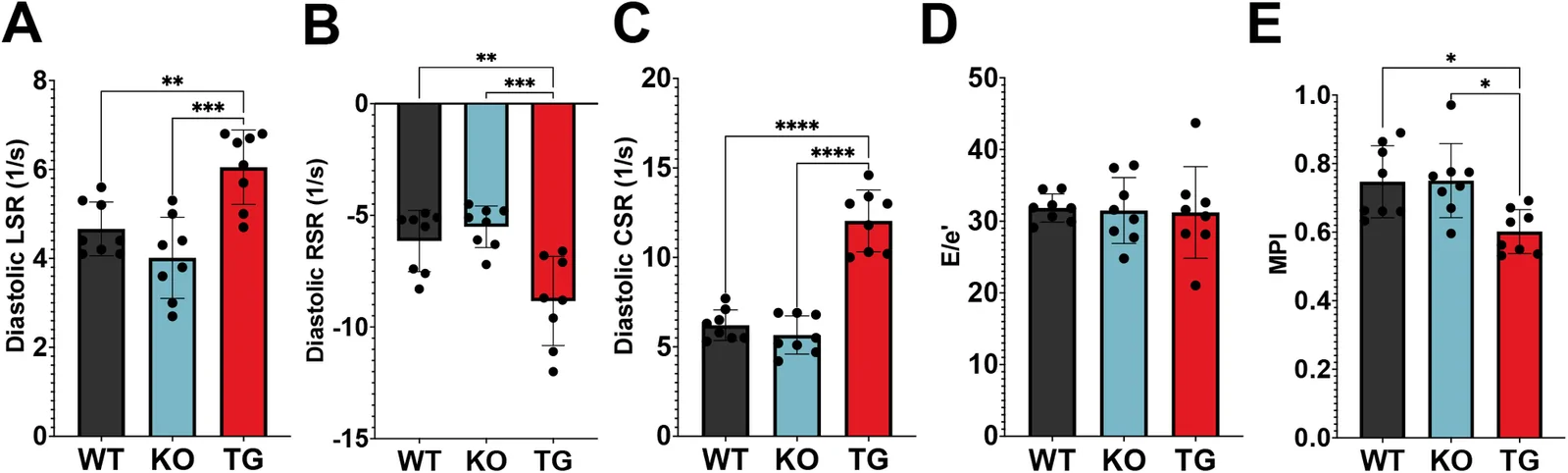
Pak1 overexpression enhances diastolic myocardial relaxation and global myocardial performance in 7-month-old mice. Representative speckle-tracking echocardiographic images for longitudinal diastolic strain rate analysis were performed in WT, Pak1 KO, and Pak1 TG mice. **(A)** Diastolic longitudinal strain rate (LSR). **(B)** Diastolic radial strain rate (RSR). **(C)** Diastolic circumferential strain rate (CSR). **(D)** Ratio of mitral inflow E velocity to early diastolic mitral annular velocity (*E*/*e*′). **(E)** Myocardial performance index (MPI). *n* = 8 per group. Data are presented as mean ± SD. Statistical comparisons were performed by one-way ANOVA with Tukey's *post hoc* test. *p < 0.05, **p < 0.01, ***p < 0.001, ****p < 0.0001.

Isovolumetric relaxation time (IVRT) was shorter in TG mice (20.13 ± 2.27 ms) than in WT (27.38 ± 3.42 ms) and KO (27.20 ± 5.57 ms) mice, while the myocardial performance index (MPI) was lower in TG mice (0.602 ± 0.06) than in WT (0.747 ± 0.11) and KO (0.751 ± 0.11) mice (overall *p* = 0.007; Figure 5E, Table 2). The lower MPI appeared primarily related to shortened isovolumetric contraction time (IVCT), whereas IVCT and aortic ejection time (AET) showed modest, nonsignificant decreases. Overall, Pak1 TG mice exhibited favorable changes in measured diastolic and global myocardial functional indices under the experimental conditions, whereas Pak1 deletion had minimal effects on diastolic indices.

### Invasive hemodynamic assessment

To complement the echocardiographic findings, invasive LV pressure catheterization was performed in a separate all-female cohort (*n* = 7 per group; Table 1). Age and body weight (BW) differed among groups: WT and TG mice were of similar age (∼11 months) and BW (∼24 g), whereas KO mice were ∼2–3 months younger and ∼2–3 g lighter than WT and TG mice. Heart rate was significantly higher in TG mice during catheterization than in WT and KO mice (*p* = 0.002), although no group difference was observed during echocardiography. The maximal rate of pressure development (+*dP*/*dt*_max_) was higher in TG mice than in KO mice (*p* = 0.021), indicating a difference in the measured index of LV systolic performance. The minimal rate of pressure decline (−*dP*/*dt*_min_) was more negative in TG mice but did not reach statistical significance (*p* = 0.236). Tau was shorter in TG than in KO mice (*p* = 0.017), while end-diastolic pressure (EDP) was lower in TG mice (*p* = 0.040). The results were consistent with higher diastolic peak strain rates observed by STE, and with the unchanged *E*/*e*′ ratio on Doppler echocardiography. End-systolic pressure and aortic blood pressure did not differ among groups. Because heart rate and loading conditions can influence these hemodynamic measures, the present findings do not establish load-independent intrinsic contractility or relaxation. Overall, WT values were generally intermediate between KO and TG mice, consistent with patterns observed by echocardiography.

### Cardiac remodeling and functional indices in aged Pak1 TG mice

To characterize the cardiac phenotype in aged Pak1 TG mice, echocardiographic assessment was performed in independent aged WT and TG cohorts (∼20–22 months old; *n* = 4 per group; Table 3). Aged TG mice exhibited higher systolic functional indices than aged WT mice, including greater FS (43.06 ± 4.16% vs. 26.30 ± 3.08%; *p* = 0.0009; Figure 6A, Table 3) and higher GLS, GRS, and GCS (all *p* < 0.005; Figures 6B–D, Table 3). Systolic peak strain rates and *s*′ were also higher in TG mice (all *p* < 0.01; Table 3). Differences in LV geometry were similarly observed, with increased RWT and LVPW thickness and reduced systolic LVID in aged TG mice. LVAW thickness and cardiomyocyte CSA were numerically higher in TG mice but did not reach statistical significance.

**Table 3:** Echocardiographic M-mode, Doppler, and speckle-tracking strain pa-rameters in aged WT and Pak1 TG mice (mean ± SD). *n* = 4 per group. Overall *P* values from Welch's *t*-test.

Parameter	WT	TG	*P* value
Baseline characteristics and morphological parameters			
Age (days)	595*.*8 ± 4*.*50	661*.*5 ± 55*.*86	0.0995
Body weight (g)	26*.*32 ± 3*.*284	27*.*40 ± 2*.*628	0.6260
CM CSA (µm^2^)	259*.*9 ± 31*.*82	296*.*5 ± 57*.*61	0.2583
CM Width (µm)			
M-mode parameters	19*.*47 ± 1*.*329	20*.*90 ± 2*.*218	0.2605
Heart rate (bpm)	382*.*6 ± 39*.*64	396*.*0 ± 46*.*15	0.06770
FS (%)	26*.*30 ± 3*.*078	43*.*06 ± 4*.*159	**0**.**0009**
LVID;s (mm)	2*.*948 ± 0*.*3361	2*.*139 ± 0*.*3184	**0**.**0130**
LVID;d (mm)	3*.*992 ± 0*.*3183	3*.*740 ± 0*.*2981	0.2929
LVAW;s (mm)	1*.*206 ± 0*.*3183	1*.*695 ± 0*.*2588	**0**.**0562**
LVAW;d (mm)	0*.*8612 ± 0*.*2709	1*.*100 ± 0*.*1878	0.2027
LVPW;s (mm)	1*.*032 ± 0*.*06597	1*.*667 ± 0*.*1064	**0**.**0002**
LVPW;d (mm)	0*.*7387 ± 0*.*1344	1*.*044 ± 0*.*1425	**0**.**0207**
RWT			
Doppler parameters	0*.*4067 ± 0*.*09853	0*.*5776 ± 0*.*08928	**0**.**0427**
*E* wave (mm/s)	536*.*6 ± 54*.*20	577*.*1 ± 86*.*94	0.4649
*A* wave (mm/s)	416*.*0 ± 121*.*4	374*.*6 ± 22*.*29	0.5481
*E*/*A*	1*.*372 ± 0*.*3861	1*.*549 ± 0*.*2878	0.4921
*e*′ (mm/s)	−19*.*44 ± 1*.*774	−18*.*88 ± 4*.*598	0.8320
*E*/*e*′	27*.*92 ± 5*.*057	31*.*86 ± 8*.*199	0.4504
*s*′ (mm/s)	15*.*81 ± 1*.*005	20*.*54 ± 1*.*255	**0**.**0013**
IVCT (ms)	18*.*23 ± 4*.*894	11*.*77 ± 1*.*635	0.1047
AET (ms)	53*.*14 ± 2*.*015	51*.*48 ± 4*.*423	0.5316
IVRT (ms)	26*.*56 ± 2*.*142	19*.*20 ± 3*.*805	**0**.**0217**
MPI	0*.*8428 ± 0*.*09237	0*.*6078 ± 0*.*1046	**0**.**0154**
Speckle-tracking echocardiography			
Global strain			
GLS (%)	−14*.*77 ± 0*.*8876	−20*.*80 ± 1*.*163	**0**.**0002**
GRS (%)	26*.*00 ± 2*.*308	39*.*14 ± 4*.*477	**0**.**0047**
GCS (%)	−23*.*09 ± 2*.*972	−35*.*43 ± 3*.*832	**0**.**0027**
Systolic peak strain rate			
Longitudinal (s^−1^)	−3*.*625 ± 0*.*3403	−6*.*225 ± 0*.*3202	*<* **0** *.* **0001**
Radial (s^−1^)	4*.*575 ± 0*.*9106	7*.*275 ± 0*.*8539	**0**.**0050**
Circumferential (s^−1^)	−5*.*950 ± 0*.*9609	−10*.*80 ± 1*.*892	**0**.**0080**
Diastolic peak strain rate			
Longitudinal (s^−1^)	4*.*475 ± 1*.*556	6*.*950 ± 1*.*580	0.0671
Radial (s^−1^)	−5*.*575 ± 1*.*936	−9*.*800 ± 1*.*140	**0**.**0139**
Circumferential (s^−1^)	7*.*300 ± 2*.*272	13*.*78 ± 1*.*959	**0**.**0052**

BW, bodyweight; VW, ventricular weight; CM, cardiomy-ocyte; CSA, mean cross-sectional area; FS, fractional shortening; LVID;s, LV internal diameter in systole; LVID;d, LV internal diameter in diastole; LVAW;s, LV anterior wall thickness in systole; LVAW;d, LV anterior wall thickness in di-astole; LVPW;s, LV posterior wall thickness in systole; LVPW;d, LV posterior wall thickness in diastole; RWT, relative wall thickness; IVCT, isovolumetric contraction time; AET, aortic ejection time; IVRT, isovolumetric relaxation time; MPI, myocardial performance index; GLS, global longitudinal strain; GRS, global radial strain; GCS, global circumferential strain.

Bold values indicate significant *P*-values from one-way ANOVA.

**Figure 6:**
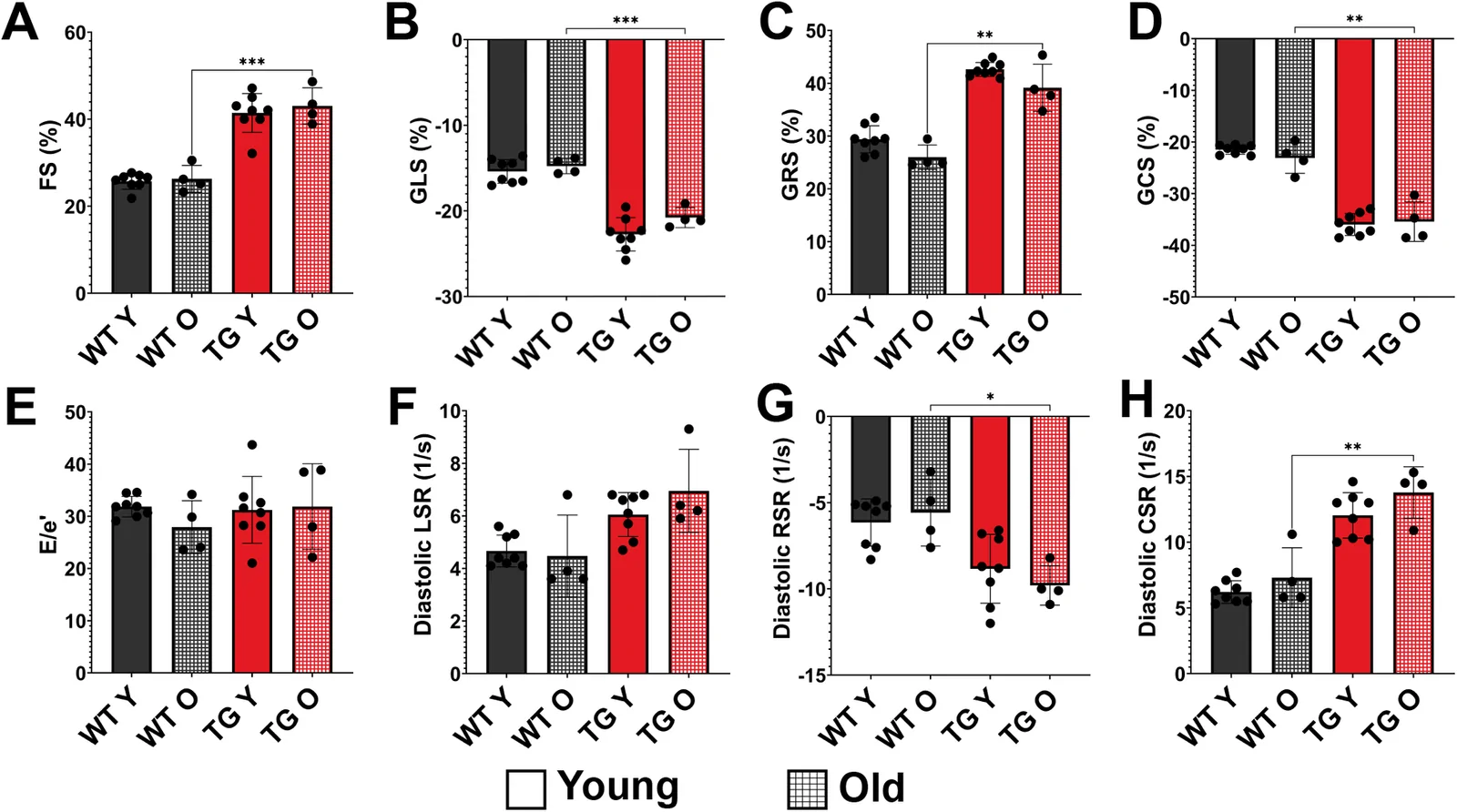
Pak1 TG mice maintained enhanced systolic and diastolic function with aging in 20–22-month-old mice. **(A)** Fractional shortening (FS). **(B)** Global longitudinal strain (GLS). **(C)** Global radial strain (GRS). **(D)** Global circumferential strain (GCS). **(E)** Ratio of mitral inflow E velocity to early diastolic mitral annular velocity (*E*/*e*′). **(F)** Diastolic longitudinal strain rate (LSR). **(G)** Diastolic radial strain rate (RSR). **(H)** Diastolic circumferential strain rate (CSR). Data are presented as mean ± SD. Statistical comparisons were performed using Welch's *t*-test, and only aged comparisons are shown. *p < 0.05, **p < 0.01, ***p < 0.001.

Diastolic functional indices also differed between groups. IVRT was shorter in aged TG mice (19.20 ± 3.81 vs. 26.56 ± 2.14 ms; *p* = 0.022), and MPI was lower (0.608 ± 0.10 vs. 0.843 ± 0.09; *p* = 0.015). Diastolic peak strain rates were higher in the circumferential and radial axes, whereas the longitudinal axis showed a nonsignificant trend (*p* = 0.067; Figures 6F–H, Table 3). *E*/*e*′ did not differ between groups (*p* = 0.45; Figure 6E, Table 3). Overall, a similar pattern of concentric remodeling and altered systolic and diastolic functional indices was observed in aged TG mice as in the younger cohort. However, because the cohorts were independent and cross-sectional, these findings do not establish preservation of function over time or protection against age-related decline.

## Discussion

This study provides a comprehensive echocardiographic and hemodynamic characterization of cardiac function in Pak1 TG mice and an assessment of baseline cardiac function in Pak1 KO mice. Using M-mode echocardiography, Doppler imaging, STE-based strain analysis, and invasive hemodynamic catheterization, we identified several differences associated with altered cardiac Pak1 expression. Cardiac-specific Pak1 overexpression was associated with increased LV wall thickness, reduced LV chamber dimensions, and higher systolic functional indices, whereas Pak1 knockout was associated with modestly lower systolic indices under baseline conditions. Pak1 TG mice also exhibited favorable changes in several diastolic functional indices. Similar differences were observed in aged TG mice. Collectively, these findings characterize an association between Pak1 expression and cardiac remodeling and increased functional indices under baseline conditions.

Pak1 TG hearts exhibited increased relative wall thickness, LVAW and LVPW thickness, and reduced LV cavity dimensions compared with WT and KO mice, consistent with a concentric LV remodeling pattern (24). Cardiomyocyte cross-sectional area and width were greater in TG than KO hearts but did not differ significantly between TG and WT hearts. Thus, these findings do not establish cardiomyocyte hypertrophy relative to WT controls. The absence of increased myocardial fibrosis, together with unchanged BNP and reduced ANP expression in TG hearts, provides no evidence of increased fibrotic or fetal-gene-expression changes under the conditions examined. The favorable functional phenotype is compatible with features of physiological remodeling; however, because the study did not directly compare TG mice with an established physiological remodeling model or assess the full spectrum of molecular markers distinguishing physiological from pathological remodeling, the phenotype should not be definitively classified as physiological remodeling. The concentric geometry resembles adaptations reported with resistance training (24, 25), although the mechanisms linking Pak1 expression to these structural changes remain to be determined.

The most prominent functional difference in Pak1 TG mice was higher systolic functional indices across multiple modalities. This pattern was evident by M-mode fractional shortening, STE-based global strain and strain rate, tissue Doppler *s*′, and invasive +*dP*/*dt*_max_. The concordance among these measurements is noteworthy because chamber-based indices such as fractional shortening can be influenced by LV geometry, particularly with concentric remodeling (23, 26). STE therefore provides complementary assessment of myocardial deformation. Invasive measurements also showed higher +*dP*/*dt*_max_ in TG mice, although heart rate was higher in the TG catheterization cohort. Thus, +*dP*/*dt*_max_ remains influenced by heart rate and loading conditions, and the present data do not establish increased intrinsic or load-independent myocardial contractility. Similarly, the lower systolic indices observed in Pak1 KO mice are consistent with an association between Pak1 deficiency and baseline systolic performance, but the magnitude and mechanism remain uncertain. Future studies using load-independent approaches, such as pressure-volume loop analysis or isolated cardiomyocytes, will be needed to determine whether Pak1 directly influences intrinsic myocardial contractility.

The diastolic findings further characterized the TG phenotype. TG mice exhibited higher E-wave velocity and *e*′, increased diastolic strain rates, shorter IVRT, lower MPI, and shorter Tau, whereas *E*/*e*′ and E/A were unchanged. These findings represent favorable changes in measured diastolic functional indices under the experimental conditions. Diastolic strain rate measurements provide complementary information, as conventional Doppler measures in mice can be influenced by the high physiological heart rate, angle dependence, and difficulty resolving transmitral E and A waves. Moreover, *E*/*e*′ may not reliably identify diastolic dysfunction in some murine disease models (16). STE-derived diastolic strain rate is less dependent on Doppler angle and provides an assessment of myocardial deformation across multiple planes; it has also been reported to detect diastolic impairment with greater sensitivity than conventional Doppler measures in murine models of cardiac disease (16, 18). Nevertheless, several parameters used in the present study, including Tau and Doppler-derived indices, remain sensitive to heart rate and loading conditions and therefore should not be interpreted as definitive evidence of enhanced intrinsic myocardial relaxation. Similarly, the unchanged *E*/*e*′ ratio indicates no detected group difference in this index but does not independently establish preserved filling pressures. Thus, the present findings support an association between Pak1 overexpression and favorable measured diastolic functional indices in the setting of concentric LV remodeling, rather than demonstrating augmented intrinsic diastolic performance.

Pak1 KO mice generally exhibited lower diastolic indices than WT mice, although most differences were not statistically significant under the baseline conditions examined. Notably, Batra et al. reported diastolic dysfunction with aging in female Pak1-deficient mice, including reduced *e*′, elevated *E*/*e*′, and left atrial enlargement (8). This observation raises the possibility that the consequences of Pak1 deficiency may become more apparent with aging or physiological stress; however, aged KO mice were not available in the present study to directly test this hypothesis.

Several functional differences between Pak1 TG and WT mice were also present in the aged cohort. Aged TG mice exhibited higher fractional shortening and global strain, higher *s*′, shorter IVRT, and lower MPI than age-matched WT mice. These findings indicate that the differences associated with Pak1 overexpression remained detectable at advanced age. However, they should not be interpreted as evidence that Pak1 prevents age-related cardiac decline. The aged cohort was small, WT mice did not show a significant decline relative to younger mice, and aged KO mice were unavailable. Furthermore, the study was cross-sectional rather than longitudinal. Thus, the present data demonstrate persistence of the TG-associated phenotype at advanced age but do not establish increased resistance to age-related functional deterioration. Longitudinal studies including all genotypes and larger cohorts will be necessary to determine whether Pak1 alters the trajectory of cardiac aging.

Several mechanisms could potentially contribute to the cardiac phenotype associated with Pak1 overexpression. Previous studies have implicated PAK1 in hypertrophic and stress-response pathways, calcium handling, and contractile regulation through proteins including phospholamban, SERCA2a, and myofilament components (10, 14, 27). Our previous work also showed that PAK1 is required for growth factor-induced Akt activation in cardiomyocytes (29), providing a potential connection to pathways involved in physiological cardiac remodeling. These pathways offer plausible explanations for some of the structural and functional differences observed in TG mice. However, none was directly examined in the present study. Accordingly, these mechanisms remain hypotheses based on the observed phenotype and prior literature rather than mechanisms demonstrated here. Targeted molecular, biochemical, and genetic studies will be necessary to determine which pathways contribute to the phenotype.

The potential translational relevance of these findings should likewise be considered cautiously. Previous studies suggest that PAK1 signaling influences cardiac hypertrophic signaling, calcium homeostasis, and electrical stability, and experimental manipulation of PAK1 has been investigated in models of cardiac disease (9, 11, 15, 27–30). The present study extends these observations by showing that cardiac-specific Pak1 overexpression is associated with favorable baseline systolic and diastolic functional indices that remain detectable in aged mice. However, the present study was conducted under baseline physiological conditions and did not directly examine disease challenge or pharmacological PAK1 activation. Thus, these findings do not establish that PAK1 activation is cardioprotective or therapeutically beneficial in heart failure or cardiac aging. Nevertheless, emerging studies provide complementary evidence supporting the potential therapeutic relevance of PAK1 modulation. For example, the small-molecule PAK1 activator JB2019 was reported to reverse metabolic stress-induced cardiac dysfunction in HFpEF mice, suggesting that PAK1 activation may have therapeutic potential in this setting (31). Future studies are needed to determine whether PAK1 modulation can beneficially alter cardiac responses to pathological stress and whether such modulation can be achieved safely and effectively.

Several limitations should be considered. First, the cardiac-specific transgenic overexpression model does not distinguish developmental effects of chronic Pak1 overexpression from effects occurring in the adult heart. Second, all experiments were conducted under baseline conditions, so whether the observed phenotype confers an advantage during pathological stress remains unknown. Third, sample sizes were modest and may have limited detection of smaller differences. Fourth, sex distribution differed among cohorts, and the study was not designed or powered to determine sex-specific effects. Fifth, aged KO mice were unavailable, and the aged WT and TG cohorts were not followed longitudinally. Sixth, measurements were performed under isoflurane anesthesia, which can influence cardiac function. Seventh, heart rate differed among groups during invasive hemodynamic assessment. Because +*dP*/*dt*_max_ is heart-rate dependent and Tau can be affected by cardiac cycle length, these differences could have contributed to some hemodynamic findings. Although aortic blood pressure did not differ among groups, this does not establish equivalent loading conditions or autonomic state. Finally, histological sections were not collected at exactly identical anatomical planes because of technical and logistical constraints, which may introduce sampling variability. Cardiomyocyte measurements were based on predefined morphological criteria, but selection bias cannot be completely excluded. Importantly, the animal, rather than the individual cardiomyocyte, was the experimental unit for histological analysis.

In summary, cardiac-specific Pak1 overexpression was associated with concentric LV remodeling and favorable systolic and diastolic functional indices under baseline conditions, whereas Pak1 deficiency was associated with modestly lower systolic indices. A similar pattern was observed in aged TG mice. Although the combination of favorable functional indices and absence of increased fibrosis is compatible with a relatively favorable remodeling phenotype, the present data do not establish cardiomyocyte hypertrophy relative to WT, intrinsic or load-independent contractility, or protection from age-related cardiac decline. Likewise, the study does not establish a specific molecular mechanism or therapeutic benefit of Pak1 modulation. These findings provide a basis for further investigation of the molecular pathways through which Pak1 is associated with cardiac remodeling and functional indices and for testing whether Pak1 modulation alters cardiac responses to various physiological and pathological stresses.

## Data Availability

The datasets presented in this article are not readily available. Requests to access the datasets should be directed to Qiangrong Liang, qliangh03@nyit.edu.
