# Supplementary material for: Pak1 overexpression is associated with mild concentric cardiac remodeling and favorable systolic and diastolic functional indices in transgenic mice
Source: Front Cardiovasc Med. 2026 Sep 30;13:1858786. doi: 10.3389/fcvm.2026.1858786 (PMC13662080; doi:10.3389/fcvm.2026.1858786)

Supplemental Figure Legend

Supplemental Figure 1

PAK1 Overexpression does not increase the expression of collagen or pathological hypertrophic marker proteins in 10-month-old mice. Cardiac tissues from were analyzed by Western blots. (A) Comparison between PAK1-TG and WT mice. (B) Comparison between PAK1-KO and WT mice. Representative immunoblots are shown at the top, with corresponding quantitative analyses below. α-Actinin, GAPDH, and Ponceau staining were used as loading controls. $n = 6$ per group. Data are presented as mean ± SD. Statistical comparisons were performed using one-way ANOVA followed by Tukey's post hoc test. *$P < 0.05$; ****$P < 0.0001$.

Supplemental Figure 2

M-mode echocardiography in 7-month-old mice reveals a concentric LV geometric phenotype in Pak1 transgenic (TG) mice. Shown are representative M-mode echocardiographic tracings from WT (top), TG (lower left), and KO (lower right) mouse hearts.

Supplemental Figure 3

Pak1 overexpression enhances systolic function in 7-month-old mice. Representative speckle-tracking echocardiographic images for longitudinal strain analysis are shown for WT (left), Pak1 KO (middle), and Pak1 TG (right) mice.

Supplemental Figure 4

Pak1 overexpression enhances diastolic myocardial relaxation and global myocardial performance in 7-month-old mice. Representative speckle-tracking echocardiographic images for longitudinal diastolic strain rate analysis are shown for WT (left), Pak1 KO (middle), and Pak1 TG (right) mice.

Supplemental Figure 1

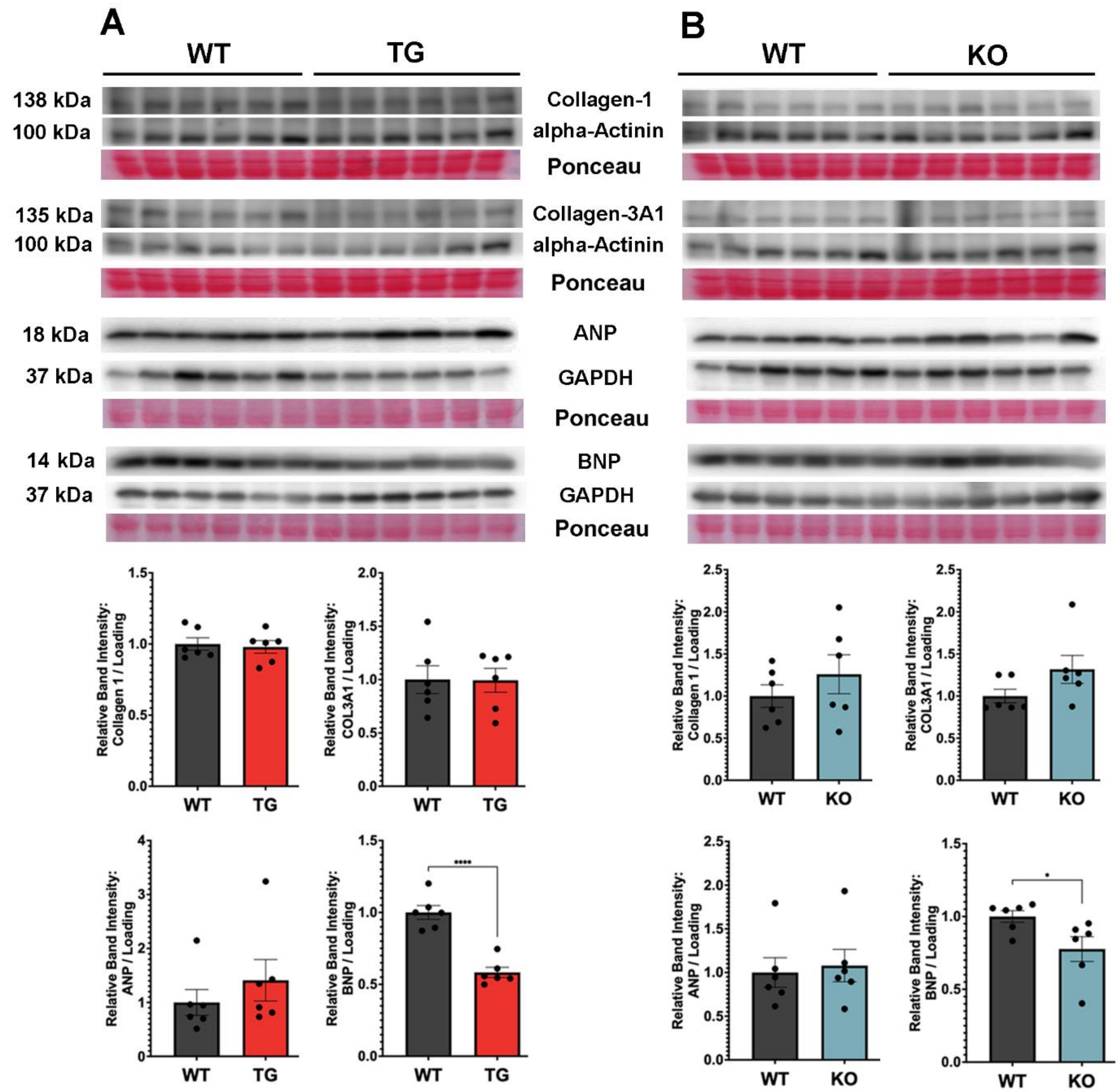

Supplemental Figure 2

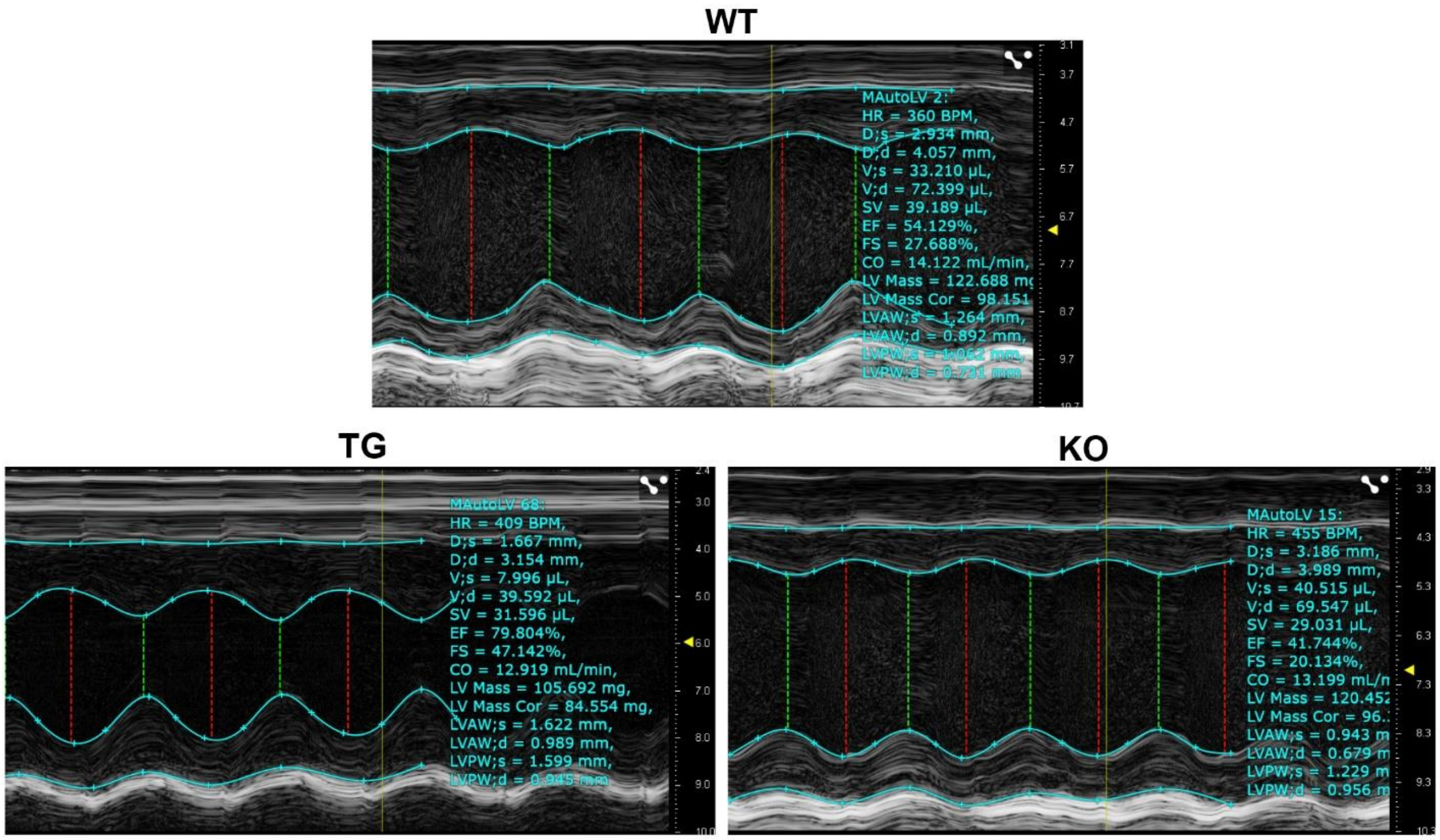

Supplemental Figure 3

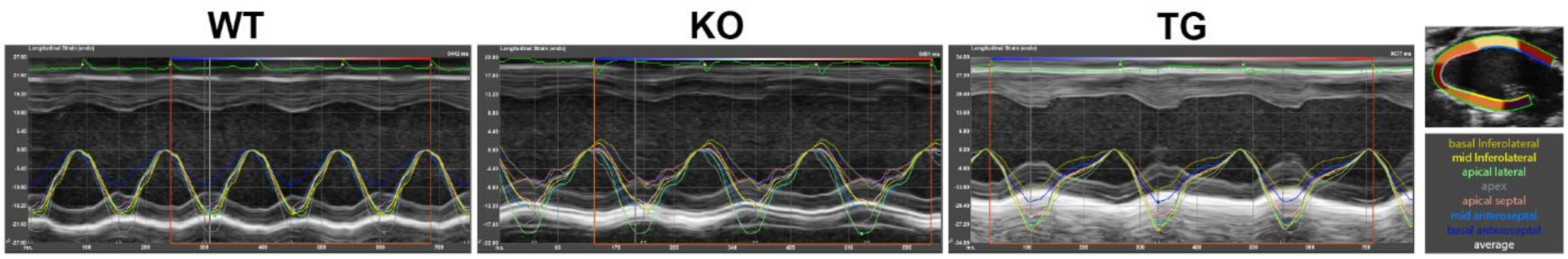

Supplemental Figure 4

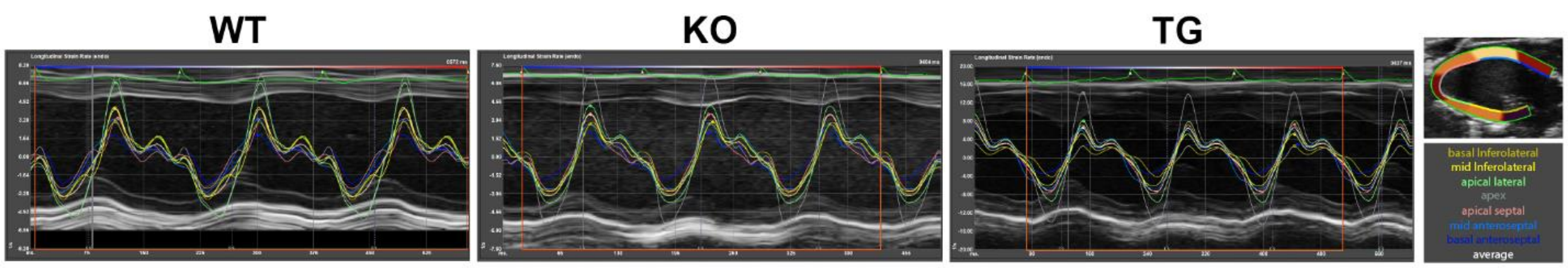

Supplement: Supplementary file 1 [file Datasheet1.pdf]
