# Supplementary material for: Pak1 overexpression is associated with mild concentric cardiac remodeling and favorable systolic and diastolic functional indices in transgenic mice
Source: Front Cardiovasc Med. 2026 Sep 30;13:1858786. doi: 10.3389/fcvm.2026.1858786 (PMC13662080; doi:10.3389/fcvm.2026.1858786)
Supplement: Supplementary file 2 [file Datasheet2.pdf]

Reagents used in this study

Reagent / Product	Manufacturer	Cat. #	Used for Buffer / Experiment
Tris	RPI	T60040	Gel / Running Buffer / Transfer Buffer / Loading Buffer
SDS	RPI	L22040	Gel / Running Buffer / Loading Buffer
Glycine	RPI	G36050	Running Buffer / Transfer Buffer
10× TBS	RPI	T60075	1× TBST / BSA / Milk
10% Tween 20 Solution	Bio-Rad	1610781	1× TBST / BSA / Milk
30% Acrylamide	Bio-Rad	1610156	Gel
TEMED	Sigma-Aldrich	T9281	Gel
APS (Ammonium Persulfate)	Sigma-Aldrich	A3678	Gel
Glycerol	Sigma-Aldrich	G5516	SDS Sample Loading Buffer
1-Thioglycerol	MCE	HY-WO10572	SDS Sample Loading Buffer
Bromophenol Blue	Sigma-Aldrich	B6131	SDS Sample Loading Buffer
BSA (Bovine Serum Albumin)	Millipore Sigma	A9647	2.5% BSA Blocking Buffer
Blotting-Grade Blocker (Nonfat Dry Milk)	Bio-Rad	1706404	2.5% Milk Blocking Buffer
10× Cell Lysis Buffer	Cell Signaling Technology	9803S	Cell Lysis Buffer
Halt Protease Inhibitor Cocktail (100×)	Thermo Fisher Scientific	78447	Cell Lysis Buffer
Phosphate Inhibitor Cocktail 3	Sigma-Aldrich	P0044	Cell Lysis Buffer

Primary antibodies and Secondary antibodies used in this study

1st / 2nd	Target	Host	Clonality / Type	Vendor	Catalog #	RRID	Working Dilution
1st	PAK1	Rabbit	Polyclonal	Cell Signaling Technology	CS2602	AB_330222	1:1,000
1st	Collagen I	Mouse	Monoclonal, IgG1	Thermo Fisher Scientific	MA1-26771	AB_2081889	1:5,000
1st	Collagen III (COL3A1)	Mouse	Monoclonal, IgG1κ	Santa Cruz Biotechnology	sc-271249		1:5,000
1st	ANP	Mouse	Monoclonal, IgG1κ	Santa Cruz Biotechnology	sc-515701	AB_3076640	1:5,000
1st	BNP	Rabbit	Polyclonal, IgG	Thermo Fisher Scientific	PA5-96084	AB_2807886	1:5,000
1st	α-Actinin	Rabbit	Polyclonal	Cell Signaling Technology	CS3134	AB_2223798	1:5,000
2nd	Anti-Rabbit IgG-HRP	Goat	Anti-Rabbit IgG	ABclonal	AS014		1:10,000
2nd	Anti-Mouse IgG-HRP	Horse	Anti-Mouse IgG	Cell Signaling Technology	CS7076	AB_330924	1:5,000
